# Supplementary material for: The Systems Biology Research Tool: evolvable open-source software
Source: BMC Syst Biol. 2008 Jun 29;2:55. doi: 10.1186/1752-0509-2-55 (PMC2446383; doi:10.1186/1752-0509-2-55)
Supplement: Additional file 1 — SBRT Archive. An archive of the current version of the Systems Biology Research Tool. [file 1752-0509-2-55-S1.zip › sbrt-1.4.0/doc/developers_guide/api/sbrt/shell/io/class-use/SbrtFile.html]

Uses of Interface sbrt.shell.io.SbrtFile


|  |  |  |
| --- | --- | --- |
| **Overview** \| **Package** \| **Class** \| **Use** \| **Tree** \| **Deprecated** \| **Index** \| **Help** | |  |
| PREV   NEXT | **FRAMES**    **NO FRAMES**     **All Classes** |  |


---


## **Uses of Interface sbrt.shell.io.SbrtFile**

| Packages that use SbrtFile | |
| --- | --- |
| **sbrt.shell.io** | Provides classes and interfaces for reading and writing files. |
| **sbrt.shell.io.fba** | Provides classes and interfaces for reading and writing files relevant to Flux Balance Analysis. |
| **sbrt.shell.mng** | Provides classes and interfaces for creating process managers. |

Uses of SbrtFile in sbrt.shell.io

| Subinterfaces of SbrtFile in sbrt.shell.io | |
| --- | --- |
| `interface` | `InputFile<E>`             This interface is used to represent input files. |
| `interface` | `MapInputFile<K,V>`             This interface is used to represent input files that contain maps. |
| `interface` | `MapOutputFile<K,V>`             This interface is used to represent output files that maps can be written to. |
| `interface` | `MapTextFile<K,V>`             This interface is used to represent text-based files that contain maps. |
| `interface` | `MapTextInputFile<K,V>`             This interface is used to represent text-based input files that contain maps. |
| `interface` | `MapTextOutputFile<K,V>`             This interface is used to represent output files that maps can be written to as text. |
| `interface` | `OutputFile<T>`             This interface is used to represent output files. |

| Classes in sbrt.shell.io that implement SbrtFile | |
| --- | --- |
| `class` | `AbstractMapInputFile<K,V>`             This abstract class is a skeleton implemenation of text-based map input files. |
| `class` | `AbstractMapOutputFile<K,V>`             This abstract class is a skeleton implementation of `MapTextOutputFile`. |
| `class` | `ComiFile<K,V>`             This class is used to read column-oriented map input files. |
| `class` | `ComoFile<K,V>`             This class is used to write maps to text files in a column-oriented way. |
| `class` | `ProcessInputFile`             This class is used to represent process input files. |
| `class` | `RomiFile<K,V>`             This class is used to read row-oriented map input files. |
| `class` | `RomoFile<K,V>`             This class is used to write maps to text files in a row-oriented way. |
| `class` | `SerializedInputFile<E>`             This class is used to read input files containing serialized objects. |
| `class` | `SerializedMapInputFile<K,V>`             This class is used to read input files containing serialized maps. |
| `class` | `SerializedOutputFile<E>`             This class is used to write serialized objects to output files. |
| `class` | `SmoFile<K,V>`             This class is used to write serialized maps to output files. |
| `class` | `TextInputFile<E>`             This class is used to read input files containing text. |
| `class` | `TextOutputFile<T>`             This class is used to represent output files containing plain text. |

Uses of SbrtFile in sbrt.shell.io.fba

| Subinterfaces of SbrtFile in sbrt.shell.io.fba | |
| --- | --- |
| `interface` | `FbaOptOutputFile`             This interface is used to represent FBA optimization output files. |

| Classes in sbrt.shell.io.fba that implement SbrtFile | |
| --- | --- |
| `class` | `ColumnOrFbaOptOutputFile`             This class is used to write column-oriented output files containing FBA optimization data. |
| `class` | `FluxCapInputFile`             This class is used to read files containing flux caps. |
| `class` | `FluxIntervalInputFile`             This class is used to read flux interval input files. |
| `class` | `RowOrFbaOptOutputFile`             This class is used to write row-oriented output files containing FBA optimization data. |
| `class` | `RxnNamExprSetInputFile<E extends MathExpr<S>,S>`             This class is used to read files containing a set of reaction name expressions. |
| `class` | `SerFbaOptOutputFile`             This class is used to write output files containing FBA optimization data as serialized maps. |

Uses of SbrtFile in sbrt.shell.mng

| Methods in sbrt.shell.mng with parameters of type SbrtFile | |
| --- | --- |
| `static void` | `AbstractProcessManager.closeFile(SbrtFile file, java.io.PrintWriter out)`             Closes the provided results manager and prints a descriptive message to the provided print writer. |

---


|  |  |  |
| --- | --- | --- |
| **Overview** \| **Package** \| **Class** \| **Use** \| **Tree** \| **Deprecated** \| **Index** \| **Help** | |  |
| PREV   NEXT | **FRAMES**    **NO FRAMES**     **All Classes** |  |


---
